# 3,3,3′,3′-Tetra­methyl-6,6′-bis­[(pyridin-4-yl)meth­oxy]-1,1′-spiro­biindane ­monohydrate

**DOI:** 10.1107/S1600536812021289

**Published:** 2012-05-16

**Authors:** Ya-Jie Zhang, Yan Sun, Shu-Mei Gao, Xiao-Qing Jiang, Yu-Heng Deng

**Affiliations:** a Department of Chemistry, Capital Normal University, Beijing 100048, People’s Republic of China

## Abstract

The asymmetric unit in the title compound, C_33_H_34_N_2_O_2_·H_2_O, consists of a V-shaped mol­ecule and a water mol­ecule to which it is hydrogen bonded. The angle between the mean planes of the two spiro-connected indane groups is 77.06 (5)°. The two five-membered rings of the indane groups have envelope conformations with the methyl­ene atoms adjacent to the spiro C atom forming the flaps. They have deviations from the mean plane of the other four atoms in the rings of 0.374 (4) and 0.362 (4) Å. In the crystal, molecules are linked to form inversion dimers *via* O—H⋯N hydrogen bonds involving the pyridine N atoms and the solvent water mol­ecule. The dimers are linked into a chain along the *b* axis by π–π stacking inter­actions between a pyridine ring and its centrosymmetrically related ring in an adjacent dimer. The centroid–centroid distance between the planes is 3.7756 (17) Å, the perpendicular distance is 3.4478 (11) Å and the offset is 1.539 Å.

## Related literature

For the use of spirane derivatives in ligand design, see: Chan *et al.* (1997 ▶); Cottam & Steel (2009 ▶); Ding *et al.* (2009 ▶); Srivastava *et al.* (1992 ▶). For 1,1′-spiro­biindane and its analogues, see: Cottam & Steel (2009 ▶); Birman *et al.* (1999 ▶); Brewster & Prudence (1973 ▶). For the experimental procedure, see: Cottam & Steel (2009 ▶); Kendhale *et al.* (2008 ▶); Yao *et al.* (2010 ▶).
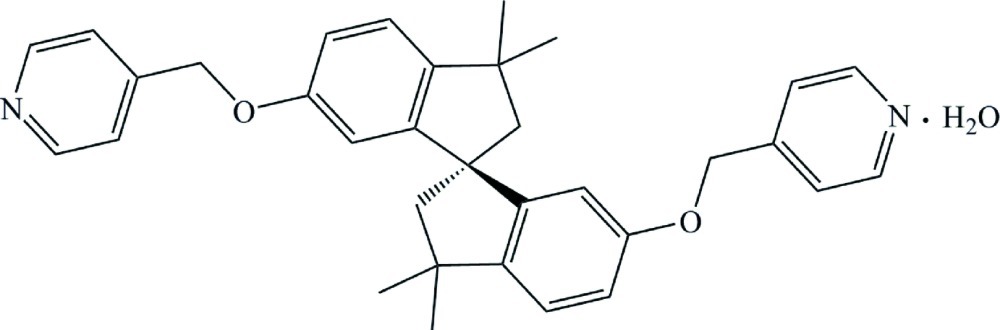


## Experimental

### 

#### Crystal data

C_33_H_34_N_2_O_2_·H_2_O
*M* *_r_* = 508.64
Triclinic, 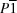

*a* = 6.0101 (12) Å
*b* = 10.724 (2) Å
*c* = 22.156 (4) Å
α = 81.92 (3)°
β = 87.17 (3)°
γ = 77.22 (3)°
*V* = 1378.6 (5) Å^3^
*Z* = 2
Mo *K*α radiation
μ = 0.08 mm^−1^
*T* = 173 K
0.49 × 0.25 × 0.21 mm

#### Data collection

Bruker APEXII CCD diffractometer
Absorption correction: multi-scan (*SADABS*; Bruker, 2007 ▶) *T*_min_ = 0.963, *T*_max_ = 0.984
10554 measured reflections
4988 independent reflections
4253 reflections with *I* > 2σ(*I*)
*R*_int_ = 0.034

#### Refinement

*R*[*F* ^2^ > 2σ(*F* ^2^)] = 0.069
*wR*(*F* ^2^) = 0.137
*S* = 1.16
4988 reflections
347 parameters
H-atom parameters constrained
Δρ_max_ = 0.24 e Å^−3^
Δρ_min_ = −0.17 e Å^−3^

### 

Data collection: *APEX2* (Bruker, 2007 ▶); cell refinement: *APEX2* and *SAINT* (Bruker, 2007 ▶); data reduction: *APEX2* and *SAINT*; program(s) used to solve structure: *SHELXS97* (Sheldrick, 2008 ▶); program(s) used to refine structure: *SHELXL97* (Sheldrick, 2008 ▶); molecular graphics: *DIAMOND* (Brandenburg, 2001 ▶); software used to prepare material for publication: *SHELXTL* (Sheldrick, 2008 ▶).

## Supplementary Material

Crystal structure: contains datablock(s) global, I. DOI: 10.1107/S1600536812021289/go2055sup1.cif

Structure factors: contains datablock(s) I. DOI: 10.1107/S1600536812021289/go2055Isup2.hkl

Supplementary material file. DOI: 10.1107/S1600536812021289/go2055Isup3.cml

Additional supplementary materials:  crystallographic information; 3D view; checkCIF report

## Figures and Tables

**Table 1:** Hydrogen-bond geometry (Å, °)

*D*—H⋯*A*	*D*—H	H⋯*A*	*D*⋯*A*	*D*—H⋯*A*
O3—H3*A*⋯N2	0.89	2.00	2.888 (3)	173
O3—H3*B*⋯N1^i^	0.89	2.05	2.940 (3)	173

Symmetry code: (i) 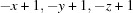.

**Fig. 1.**
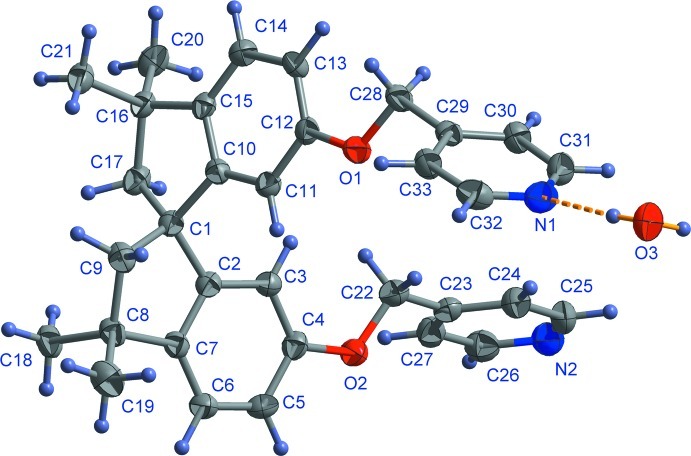
ORTEP drawing of title compound with the atom-numbering scheme, showing 30% probability displacement ellipsoids. (All labels of the H atoms are omitted for clarity, the dotted line represents as hydrogen bond).

**Fig. 2.**
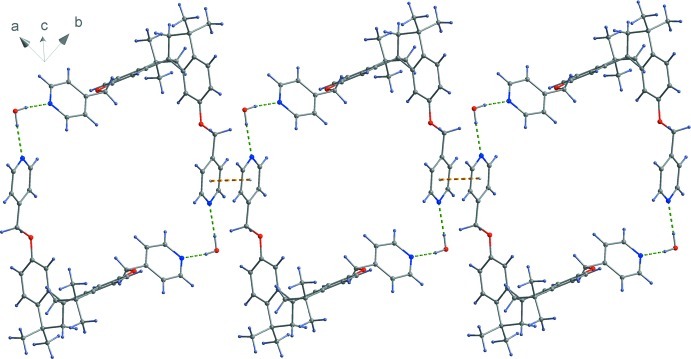
The chain structure constructed by the dimers by the π—π stacking forces shown as dotted lines.

## supplementary crystallographic information

### Comment

Spirane derivatives, which are typical molecules with axial chirality, have been mainly employed in ligand design and asymmetric synthesis (Srivastava *et al.*, 1992; Chan *et al.*, 1997; Ding *et al.*, 2009; Cottam & Steel, 2009). Among them, 1,1'-spirobiindane and its analogs have also attracted much attention for their featuring C2-symmetric chiral property (Birman *et al.*, 1999; Brewster & Prudence, 1973; Cottam & Steel, 2009). Following our previous work(Yao *et al.*, 2010), we report the structure of a bidentate flexible nitrogen ligand by coupling 1,1,-spirobiindane with nitrogen-containing heterocycles.

The molecule of C_33_H_34_N_2_O_2_.H_2_O, is V-shaped, Figure 1. The angle between the mean planes of the two spiro connected indane groups is 77.06 (5)°.

The two five membered rings of the indane groups have an envelope pucker with the methylene atoms, C9 and C17, adjacent to the spiro carbon atom forming the flaps. They have deviations from mean plane of other 4 atoms in the rings of 0.374 (4) Å and 0.362 (4) Å, respectively.

Two molecules are linked to form a centrosymmetric dimer *via* hydrogen bonds between the pyridinyl nitrogen atoms and the solvent water, O3—H3A···N2 (within the asymmetric unit) and O3—H3B···N1 (1 - *x*,1 - *y*,1 - *z*), Table 1 and Figure 2.

The dimers are linked to form a one-dimensional chain structure by π—π stacking interactions between the pyridinyl ring (C29—C30—N1—C31—C32—C33) and its centrosymmetrically related ring at (3 - *x*,-*y*,1 - *z*+). The centroid-centroid distance between the planes is 3.7756 (17) Å, the perpendicular distance is 3.4478 (11) Å and the offset is 1.539 Å, Figure 2.

### Experimental

The starting material compound, 6,6'-dihydroxy-3,3,3',3'-tetramethyl- 1,1'-spirobiindane, was synthesized by the literature method (Kendhale *et al.*, 2008). The title compound was prepared following the literature procedure (Cottam & Steel, 2009). A mixture of 6,6'-dihydroxy-3,3,3',3'- tetramethyl-1,1'-spirobiindane, 4-chloromethylpyridine and sodium hydroxide (mole ratio: 1:2:4) was refluxed in dry acetone solvent for 3 days under an N_2_ atmosphere. The solid crude product was obtained by removal of the solvent and further purified by column chromatography on silica gel (hexane/EtOAc = 8:2 v.v), yield 50%. Colourless crystals were obtained by slow evaporation from a solution of hexane.

### Refinement

The hydrogen atoms were placed in idealized positions and allowed to ride on the relevant atoms, with C—H = 0.93 and 0.97 Å for aryl and methylene H atoms, respectively, U*_iso_*(H)=1.2U*_eq_*(C), O—H = 0.89 Å with U*_iso_*(H)=1.2U*_eq_*(O). The positions of the methyl and water H atoms were checked on a final difference Fourier.

### Crystal data

**Table:**

C_33_H_34_N_2_O_2_·H_2_O	*Z* = 2
*M**_r_* = 508.64	*F*(000) = 544
Triclinic, *P*1	*D*_x_ = 1.225 Mg m^−^^3^
*a* = 6.0101 (12) Å	Mo *K*α radiation, λ = 0.71073 Å
*b* = 10.724 (2) Å	Cell parameters from 3702 reflections
*c* = 22.156 (4) Å	θ = 1.9–25.3°
α = 81.92 (3)°	µ = 0.08 mm^−^^1^
β = 87.17 (3)°	*T* = 173 K
γ = 77.22 (3)°	Block, colourless
*V* = 1378.6 (5) Å^3^	0.49 × 0.25 × 0.21 mm

### Data collection

**Table:**

Bruker APEXII CCD diffractometer	4988 independent reflections
Radiation source: fine-focus sealed tube	4253 reflections with *I* > 2σ(*I*)
Graphite monochromator	*R*_int_ = 0.034
φ and ω scans	θ_max_ = 25.3°, θ_min_ = 2.9°
Absorption correction: multi-scan (*SADABS*; Bruker, 2007)	*h* = −7→7
*T*_min_ = 0.963, *T*_max_ = 0.984	*k* = −12→12
10554 measured reflections	*l* = −24→26

### Refinement

**Table:**

Refinement on *F*^2^	Primary atom site location: structure-invariant direct methods
Least-squares matrix: full	Secondary atom site location: difference Fourier map
*R*[*F*^2^ > 2σ(*F*^2^)] = 0.069	Hydrogen site location: inferred from neighbouring sites
*wR*(*F*^2^) = 0.137	H-atom parameters constrained
*S* = 1.16	*w* = 1/[σ^2^(*F*_o_^2^) + (0.035*P*)^2^ + 0.7601*P*] where *P* = (*F*_o_^2^ + 2*F*_c_^2^)/3
4988 reflections	(Δ/σ)_max_ < 0.001
347 parameters	Δρ_max_ = 0.24 e Å^−^^3^
0 restraints	Δρ_min_ = −0.17 e Å^−^^3^

### Special details

**Table:**

Geometry. All e.s.d.'s (except the e.s.d. in the dihedral angle between two l.s. planes) are estimated using the full covariance matrix. The cell e.s.d.'s are taken into account individually in the estimation of e.s.d.'s in distances, angles and torsion angles; correlations between e.s.d.'s in cell parameters are only used when they are defined by crystal symmetry. An approximate (isotropic) treatment of cell e.s.d.'s is used for estimating e.s.d.'s involving l.s. planes.
Refinement. Refinement of *F*^2^ against ALL reflections. The weighted *R*-factor *wR* and goodness of fit *S* are based on *F*^2^, conventional *R*-factors *R* are based on *F*, with *F* set to zero for negative *F*^2^. The threshold expression of *F*^2^ > σ(*F*^2^) is used only for calculating *R*-factors(gt) *etc*. and is not relevant to the choice of reflections for refinement. *R*-factors based on *F*^2^ are statistically about twice as large as those based on *F*, and *R*- factors based on ALL data will be even larger.

### Fractional atomic coordinates and isotropic or equivalent isotropic displacement parameters (Å^2^)

**Table:**

	*x*	*y*	*z*	*U*_iso_*/*U*_eq_	
O1	1.1312 (3)	0.04027 (16)	0.33047 (7)	0.0397 (4)	
O2	0.1540 (3)	0.43323 (16)	0.23137 (8)	0.0427 (4)	
N1	1.5601 (4)	0.2248 (2)	0.46452 (11)	0.0507 (6)	
N2	−0.4528 (4)	0.5560 (2)	0.38411 (11)	0.0528 (6)	
C1	0.6971 (4)	0.0684 (2)	0.14537 (10)	0.0306 (5)	
C2	0.5655 (4)	0.2053 (2)	0.14847 (10)	0.0292 (5)	
C3	0.4135 (4)	0.2482 (2)	0.19451 (10)	0.0314 (5)	
H3	0.3835	0.1900	0.2288	0.038*	
C4	0.3069 (4)	0.3778 (2)	0.18911 (11)	0.0336 (5)	
C5	0.3525 (4)	0.4625 (2)	0.13868 (11)	0.0395 (6)	
H5	0.2780	0.5511	0.1353	0.047*	
C6	0.5050 (4)	0.4188 (2)	0.09374 (11)	0.0381 (6)	
H6	0.5361	0.4771	0.0596	0.046*	
C7	0.6129 (4)	0.2894 (2)	0.09859 (10)	0.0313 (5)	
C8	0.7834 (4)	0.2204 (2)	0.05474 (11)	0.0351 (6)	
C9	0.8772 (4)	0.0906 (2)	0.09442 (11)	0.0386 (6)	
H9A	1.0229	0.0928	0.1128	0.046*	
H9B	0.9059	0.0194	0.0691	0.046*	
C10	0.8030 (4)	0.0020 (2)	0.20557 (10)	0.0286 (5)	
C11	0.9276 (4)	0.0530 (2)	0.24253 (10)	0.0313 (5)	
H11	0.9555	0.1370	0.2312	0.038*	
C12	1.0119 (4)	−0.0205 (2)	0.29660 (10)	0.0318 (5)	
C13	0.9705 (4)	−0.1428 (2)	0.31379 (10)	0.0345 (6)	
H13	1.0265	−0.1919	0.3512	0.041*	
C14	0.8451 (4)	−0.1929 (2)	0.27528 (11)	0.0350 (5)	
H14	0.8170	−0.2770	0.2864	0.042*	
C15	0.7615 (4)	−0.1208 (2)	0.22100 (10)	0.0291 (5)	
C16	0.6323 (4)	−0.1574 (2)	0.17100 (11)	0.0315 (5)	
C17	0.5423 (4)	−0.0241 (2)	0.13356 (11)	0.0373 (6)	
H17A	0.3828	0.0110	0.1460	0.045*	
H17B	0.5455	−0.0327	0.0896	0.045*	
C18	0.6609 (4)	0.2012 (3)	−0.00128 (11)	0.0429 (6)	
H18A	0.5397	0.1550	0.0119	0.064*	
H18B	0.7705	0.1510	−0.0277	0.064*	
H18C	0.5941	0.2855	−0.0239	0.064*	
C19	0.9733 (4)	0.2922 (3)	0.03409 (13)	0.0521 (7)	
H19A	0.9085	0.3739	0.0091	0.078*	
H19B	1.0881	0.2390	0.0100	0.078*	
H19C	1.0449	0.3095	0.0699	0.078*	
C20	0.4349 (4)	−0.2201 (3)	0.19443 (13)	0.0433 (6)	
H20A	0.4950	−0.3051	0.2172	0.065*	
H20B	0.3473	−0.2297	0.1599	0.065*	
H20C	0.3354	−0.1656	0.2213	0.065*	
C21	0.7993 (4)	−0.2496 (2)	0.13409 (12)	0.0405 (6)	
H21A	0.9238	−0.2087	0.1176	0.061*	
H21B	0.7186	−0.2695	0.1005	0.061*	
H21C	0.8619	−0.3295	0.1607	0.061*	
C22	0.1231 (4)	0.3548 (2)	0.28763 (12)	0.0405 (6)	
H22A	0.2627	0.3366	0.3122	0.049*	
H22B	0.0926	0.2717	0.2796	0.049*	
C23	−0.0741 (4)	0.4264 (2)	0.32137 (11)	0.0361 (6)	
C24	−0.0538 (5)	0.4563 (2)	0.37921 (12)	0.0445 (6)	
H24	0.0898	0.4334	0.3986	0.053*	
C25	−0.2440 (5)	0.5196 (3)	0.40854 (13)	0.0533 (8)	
H25	−0.2268	0.5388	0.4484	0.064*	
C26	−0.4693 (5)	0.5269 (3)	0.32831 (13)	0.0479 (7)	
H26	−0.6144	0.5520	0.3098	0.057*	
C27	−0.2893 (4)	0.4629 (2)	0.29563 (12)	0.0410 (6)	
H27	−0.3119	0.4439	0.2561	0.049*	
C28	1.2225 (4)	−0.0277 (2)	0.38685 (11)	0.0400 (6)	
H28A	1.3343	−0.1076	0.3800	0.048*	
H28B	1.0993	−0.0513	0.4144	0.048*	
C29	1.3368 (4)	0.0620 (2)	0.41402 (10)	0.0336 (5)	
C30	1.2521 (4)	0.1158 (2)	0.46561 (11)	0.0388 (6)	
H30	1.1150	0.0987	0.4847	0.047*	
C31	1.3682 (5)	0.1949 (3)	0.48926 (12)	0.0473 (7)	
H31	1.3082	0.2300	0.5252	0.057*	
C32	1.6353 (4)	0.1750 (3)	0.41367 (13)	0.0463 (7)	
H32	1.7691	0.1965	0.3944	0.056*	
C33	1.5327 (4)	0.0949 (3)	0.38721 (11)	0.0404 (6)	
H33	1.5951	0.0623	0.3509	0.048*	
O3	−0.8355 (3)	0.67390 (19)	0.45596 (8)	0.0520 (5)	
H3A	−0.7253	0.6347	0.4324	0.078*	
H3B	−0.7606	0.7035	0.4826	0.078*	

### Atomic displacement parameters (Å^2^)

**Table:**

	*U*^11^	*U*^22^	*U*^33^	*U*^12^	*U*^13^	*U*^23^
O1	0.0548 (11)	0.0366 (10)	0.0309 (9)	−0.0167 (8)	−0.0149 (8)	−0.0001 (7)
O2	0.0518 (11)	0.0325 (10)	0.0368 (10)	0.0030 (8)	0.0097 (8)	−0.0040 (8)
N1	0.0622 (15)	0.0435 (14)	0.0499 (15)	−0.0175 (11)	−0.0158 (12)	−0.0026 (11)
N2	0.0571 (15)	0.0490 (15)	0.0481 (15)	−0.0058 (12)	0.0146 (12)	−0.0065 (11)
C1	0.0310 (12)	0.0293 (12)	0.0299 (12)	−0.0006 (9)	−0.0058 (10)	−0.0058 (10)
C2	0.0268 (11)	0.0292 (12)	0.0311 (12)	−0.0030 (9)	−0.0053 (9)	−0.0060 (10)
C3	0.0330 (12)	0.0294 (12)	0.0307 (13)	−0.0053 (10)	−0.0019 (10)	−0.0024 (10)
C4	0.0308 (12)	0.0335 (13)	0.0355 (13)	−0.0022 (10)	0.0014 (10)	−0.0092 (10)
C5	0.0460 (15)	0.0266 (13)	0.0422 (15)	−0.0014 (11)	−0.0023 (12)	−0.0015 (11)
C6	0.0452 (14)	0.0313 (13)	0.0353 (14)	−0.0066 (11)	0.0014 (11)	0.0003 (11)
C7	0.0313 (12)	0.0330 (13)	0.0286 (12)	−0.0037 (10)	−0.0051 (10)	−0.0042 (10)
C8	0.0330 (13)	0.0406 (14)	0.0286 (13)	−0.0023 (10)	−0.0023 (10)	−0.0022 (10)
C9	0.0325 (13)	0.0450 (15)	0.0321 (13)	0.0044 (11)	−0.0018 (10)	−0.0046 (11)
C10	0.0261 (11)	0.0300 (12)	0.0269 (12)	0.0005 (9)	−0.0015 (9)	−0.0042 (9)
C11	0.0332 (12)	0.0293 (12)	0.0315 (13)	−0.0060 (10)	−0.0040 (10)	−0.0044 (10)
C12	0.0343 (12)	0.0332 (13)	0.0294 (13)	−0.0078 (10)	−0.0020 (10)	−0.0085 (10)
C13	0.0404 (14)	0.0376 (14)	0.0238 (12)	−0.0065 (11)	−0.0035 (10)	−0.0004 (10)
C14	0.0386 (13)	0.0309 (13)	0.0359 (14)	−0.0091 (10)	−0.0003 (11)	−0.0035 (10)
C15	0.0252 (11)	0.0293 (12)	0.0316 (12)	−0.0009 (9)	0.0002 (9)	−0.0080 (10)
C16	0.0304 (12)	0.0293 (13)	0.0350 (13)	−0.0027 (10)	−0.0040 (10)	−0.0092 (10)
C17	0.0380 (13)	0.0334 (14)	0.0405 (14)	−0.0015 (10)	−0.0132 (11)	−0.0109 (11)
C18	0.0453 (15)	0.0482 (16)	0.0295 (13)	0.0035 (12)	−0.0064 (11)	−0.0058 (11)
C19	0.0449 (16)	0.065 (2)	0.0454 (17)	−0.0152 (14)	0.0060 (13)	−0.0031 (14)
C20	0.0371 (14)	0.0431 (16)	0.0535 (17)	−0.0118 (11)	−0.0017 (12)	−0.0139 (13)
C21	0.0406 (14)	0.0403 (15)	0.0397 (15)	−0.0005 (11)	−0.0045 (11)	−0.0136 (12)
C22	0.0463 (15)	0.0314 (14)	0.0422 (15)	−0.0057 (11)	0.0005 (12)	−0.0039 (11)
C23	0.0449 (14)	0.0287 (13)	0.0369 (14)	−0.0123 (11)	0.0015 (11)	−0.0054 (10)
C24	0.0543 (16)	0.0398 (15)	0.0398 (15)	−0.0104 (12)	−0.0047 (12)	−0.0053 (12)
C25	0.078 (2)	0.0466 (17)	0.0330 (15)	−0.0080 (15)	0.0076 (14)	−0.0106 (13)
C26	0.0449 (15)	0.0467 (16)	0.0514 (17)	−0.0113 (13)	0.0031 (13)	−0.0035 (13)
C27	0.0460 (15)	0.0413 (15)	0.0387 (15)	−0.0145 (12)	0.0022 (12)	−0.0087 (12)
C28	0.0528 (16)	0.0402 (15)	0.0281 (13)	−0.0147 (12)	−0.0095 (11)	0.0019 (11)
C29	0.0383 (13)	0.0319 (13)	0.0292 (13)	−0.0058 (10)	−0.0085 (10)	0.0011 (10)
C30	0.0358 (13)	0.0449 (15)	0.0344 (14)	−0.0066 (11)	−0.0011 (11)	−0.0041 (11)
C31	0.0596 (18)	0.0447 (16)	0.0379 (15)	−0.0071 (13)	−0.0071 (13)	−0.0114 (12)
C32	0.0420 (15)	0.0523 (17)	0.0459 (16)	−0.0182 (13)	−0.0060 (12)	0.0042 (13)
C33	0.0430 (14)	0.0449 (15)	0.0329 (14)	−0.0086 (12)	0.0000 (11)	−0.0055 (11)
O3	0.0498 (11)	0.0609 (13)	0.0485 (12)	−0.0123 (9)	−0.0020 (9)	−0.0167 (10)

### Geometric parameters (Å, º)

**Table:**

O1—C12	1.378 (3)	C16—C17	1.547 (3)
O1—C28	1.424 (3)	C17—H17A	0.9900
O2—C4	1.380 (3)	C17—H17B	0.9900
O2—C22	1.431 (3)	C18—H18A	0.9800
N1—C32	1.331 (3)	C18—H18B	0.9800
N1—C31	1.336 (4)	C18—H18C	0.9800
N2—C26	1.330 (4)	C19—H19A	0.9800
N2—C25	1.345 (4)	C19—H19B	0.9800
C1—C2	1.515 (3)	C19—H19C	0.9800
C1—C10	1.519 (3)	C20—H20A	0.9800
C1—C9	1.556 (3)	C20—H20B	0.9800
C1—C17	1.556 (3)	C20—H20C	0.9800
C2—C7	1.385 (3)	C21—H21A	0.9800
C2—C3	1.393 (3)	C21—H21B	0.9800
C3—C4	1.387 (3)	C21—H21C	0.9800
C3—H3	0.9500	C22—C23	1.491 (3)
C4—C5	1.394 (3)	C22—H22A	0.9900
C5—C6	1.379 (3)	C22—H22B	0.9900
C5—H5	0.9500	C23—C24	1.381 (3)
C6—C7	1.388 (3)	C23—C27	1.392 (4)
C6—H6	0.9500	C24—C25	1.378 (4)
C7—C8	1.520 (3)	C24—H24	0.9500
C8—C19	1.531 (3)	C25—H25	0.9500
C8—C18	1.534 (3)	C26—C27	1.378 (4)
C8—C9	1.545 (3)	C26—H26	0.9500
C9—H9A	0.9900	C27—H27	0.9500
C9—H9B	0.9900	C28—C29	1.502 (3)
C10—C11	1.378 (3)	C28—H28A	0.9900
C10—C15	1.387 (3)	C28—H28B	0.9900
C11—C12	1.389 (3)	C29—C30	1.377 (3)
C11—H11	0.9500	C29—C33	1.386 (3)
C12—C13	1.387 (3)	C30—C31	1.379 (4)
C13—C14	1.399 (3)	C30—H30	0.9500
C13—H13	0.9500	C31—H31	0.9500
C14—C15	1.387 (3)	C32—C33	1.368 (4)
C14—H14	0.9500	C32—H32	0.9500
C15—C16	1.525 (3)	C33—H33	0.9500
C16—C20	1.524 (3)	O3—H3A	0.8906
C16—C21	1.536 (3)	O3—H3B	0.8904
			
C12—O1—C28	118.01 (18)	H17A—C17—H17B	108.4
C4—O2—C22	117.53 (18)	C8—C18—H18A	109.5
C32—N1—C31	115.9 (2)	C8—C18—H18B	109.5
C26—N2—C25	116.0 (2)	H18A—C18—H18B	109.5
C2—C1—C10	113.22 (19)	C8—C18—H18C	109.5
C2—C1—C9	101.17 (19)	H18A—C18—H18C	109.5
C10—C1—C9	113.16 (18)	H18B—C18—H18C	109.5
C2—C1—C17	113.07 (18)	C8—C19—H19A	109.5
C10—C1—C17	101.16 (19)	C8—C19—H19B	109.5
C9—C1—C17	115.6 (2)	H19A—C19—H19B	109.5
C7—C2—C3	121.4 (2)	C8—C19—H19C	109.5
C7—C2—C1	111.9 (2)	H19A—C19—H19C	109.5
C3—C2—C1	126.7 (2)	H19B—C19—H19C	109.5
C4—C3—C2	118.5 (2)	C16—C20—H20A	109.5
C4—C3—H3	120.8	C16—C20—H20B	109.5
C2—C3—H3	120.8	H20A—C20—H20B	109.5
O2—C4—C3	124.5 (2)	C16—C20—H20C	109.5
O2—C4—C5	115.1 (2)	H20A—C20—H20C	109.5
C3—C4—C5	120.4 (2)	H20B—C20—H20C	109.5
C6—C5—C4	120.5 (2)	C16—C21—H21A	109.5
C6—C5—H5	119.8	C16—C21—H21B	109.5
C4—C5—H5	119.8	H21A—C21—H21B	109.5
C5—C6—C7	119.7 (2)	C16—C21—H21C	109.5
C5—C6—H6	120.1	H21A—C21—H21C	109.5
C7—C6—H6	120.1	H21B—C21—H21C	109.5
C2—C7—C6	119.6 (2)	O2—C22—C23	108.06 (19)
C2—C7—C8	111.7 (2)	O2—C22—H22A	110.1
C6—C7—C8	128.7 (2)	C23—C22—H22A	110.1
C7—C8—C19	112.7 (2)	O2—C22—H22B	110.1
C7—C8—C18	110.23 (19)	C23—C22—H22B	110.1
C19—C8—C18	109.5 (2)	H22A—C22—H22B	108.4
C7—C8—C9	101.34 (18)	C24—C23—C27	117.4 (2)
C19—C8—C9	111.4 (2)	C24—C23—C22	122.2 (2)
C18—C8—C9	111.5 (2)	C27—C23—C22	120.4 (2)
C8—C9—C1	108.22 (18)	C25—C24—C23	119.3 (3)
C8—C9—H9A	110.1	C25—C24—H24	120.3
C1—C9—H9A	110.1	C23—C24—H24	120.3
C8—C9—H9B	110.1	N2—C25—C24	123.9 (3)
C1—C9—H9B	110.1	N2—C25—H25	118.0
H9A—C9—H9B	108.4	C24—C25—H25	118.0
C11—C10—C15	121.5 (2)	N2—C26—C27	124.2 (3)
C11—C10—C1	126.1 (2)	N2—C26—H26	117.9
C15—C10—C1	112.4 (2)	C27—C26—H26	117.9
C10—C11—C12	118.9 (2)	C26—C27—C23	119.1 (3)
C10—C11—H11	120.5	C26—C27—H27	120.4
C12—C11—H11	120.5	C23—C27—H27	120.4
O1—C12—C13	124.8 (2)	O1—C28—C29	106.27 (19)
O1—C12—C11	114.2 (2)	O1—C28—H28A	110.5
C13—C12—C11	121.0 (2)	C29—C28—H28A	110.5
C12—C13—C14	119.0 (2)	O1—C28—H28B	110.5
C12—C13—H13	120.5	C29—C28—H28B	110.5
C14—C13—H13	120.5	H28A—C28—H28B	108.7
C15—C14—C13	120.5 (2)	C30—C29—C33	117.3 (2)
C15—C14—H14	119.7	C30—C29—C28	122.1 (2)
C13—C14—H14	119.7	C33—C29—C28	120.6 (2)
C14—C15—C10	119.0 (2)	C29—C30—C31	119.4 (2)
C14—C15—C16	129.7 (2)	C29—C30—H30	120.3
C10—C15—C16	111.2 (2)	C31—C30—H30	120.3
C20—C16—C15	114.1 (2)	N1—C31—C30	123.8 (3)
C20—C16—C21	109.0 (2)	N1—C31—H31	118.1
C15—C16—C21	109.26 (18)	C30—C31—H31	118.1
C20—C16—C17	110.53 (19)	N1—C32—C33	124.4 (3)
C15—C16—C17	101.35 (18)	N1—C32—H32	117.8
C21—C16—C17	112.5 (2)	C33—C32—H32	117.8
C16—C17—C1	108.51 (18)	C32—C33—C29	119.2 (2)
C16—C17—H17A	110.0	C32—C33—H33	120.4
C1—C17—H17A	110.0	C29—C33—H33	120.4
C16—C17—H17B	110.0	H3A—O3—H3B	103.8
C1—C17—H17B	110.0		
			
C10—C1—C2—C7	135.5 (2)	C10—C11—C12—C13	−0.6 (3)
C9—C1—C2—C7	14.1 (2)	O1—C12—C13—C14	179.5 (2)
C17—C1—C2—C7	−110.2 (2)	C11—C12—C13—C14	1.1 (3)
C10—C1—C2—C3	−44.3 (3)	C12—C13—C14—C15	−0.6 (3)
C9—C1—C2—C3	−165.7 (2)	C13—C14—C15—C10	−0.3 (3)
C17—C1—C2—C3	70.0 (3)	C13—C14—C15—C16	176.7 (2)
C7—C2—C3—C4	0.8 (3)	C11—C10—C15—C14	0.9 (3)
C1—C2—C3—C4	−179.4 (2)	C1—C10—C15—C14	−179.16 (19)
C22—O2—C4—C3	7.4 (3)	C11—C10—C15—C16	−176.65 (19)
C22—O2—C4—C5	−172.1 (2)	C1—C10—C15—C16	3.3 (3)
C2—C3—C4—O2	−179.6 (2)	C14—C15—C16—C20	47.7 (3)
C2—C3—C4—C5	−0.2 (3)	C10—C15—C16—C20	−135.1 (2)
O2—C4—C5—C6	179.1 (2)	C14—C15—C16—C21	−74.5 (3)
C3—C4—C5—C6	−0.4 (4)	C10—C15—C16—C21	102.7 (2)
C4—C5—C6—C7	0.4 (4)	C14—C15—C16—C17	166.5 (2)
C3—C2—C7—C6	−0.8 (3)	C10—C15—C16—C17	−16.3 (2)
C1—C2—C7—C6	179.4 (2)	C20—C16—C17—C1	144.4 (2)
C3—C2—C7—C8	179.9 (2)	C15—C16—C17—C1	23.1 (2)
C1—C2—C7—C8	0.1 (3)	C21—C16—C17—C1	−93.5 (2)
C5—C6—C7—C2	0.2 (4)	C2—C1—C17—C16	−142.7 (2)
C5—C6—C7—C8	179.4 (2)	C10—C1—C17—C16	−21.3 (2)
C2—C7—C8—C19	−133.6 (2)	C9—C1—C17—C16	101.4 (2)
C6—C7—C8—C19	47.2 (3)	C4—O2—C22—C23	−170.9 (2)
C2—C7—C8—C18	103.8 (2)	O2—C22—C23—C24	−122.1 (3)
C6—C7—C8—C18	−75.4 (3)	O2—C22—C23—C27	59.6 (3)
C2—C7—C8—C9	−14.3 (3)	C27—C23—C24—C25	0.1 (4)
C6—C7—C8—C9	166.5 (2)	C22—C23—C24—C25	−178.3 (2)
C7—C8—C9—C1	22.9 (2)	C26—N2—C25—C24	0.1 (4)
C19—C8—C9—C1	143.0 (2)	C23—C24—C25—N2	−0.4 (4)
C18—C8—C9—C1	−94.3 (2)	C25—N2—C26—C27	0.5 (4)
C2—C1—C9—C8	−22.9 (2)	N2—C26—C27—C23	−0.8 (4)
C10—C1—C9—C8	−144.3 (2)	C24—C23—C27—C26	0.5 (4)
C17—C1—C9—C8	99.6 (2)	C22—C23—C27—C26	178.9 (2)
C2—C1—C10—C11	−47.6 (3)	C12—O1—C28—C29	−178.67 (19)
C9—C1—C10—C11	66.8 (3)	O1—C28—C29—C30	111.0 (2)
C17—C1—C10—C11	−168.9 (2)	O1—C28—C29—C33	−68.3 (3)
C2—C1—C10—C15	132.4 (2)	C33—C29—C30—C31	−2.3 (3)
C9—C1—C10—C15	−113.2 (2)	C28—C29—C30—C31	178.3 (2)
C17—C1—C10—C15	11.2 (2)	C32—N1—C31—C30	1.1 (4)
C15—C10—C11—C12	−0.4 (3)	C29—C30—C31—N1	0.9 (4)
C1—C10—C11—C12	179.6 (2)	C31—N1—C32—C33	−1.7 (4)
C28—O1—C12—C13	1.1 (3)	N1—C32—C33—C29	0.2 (4)
C28—O1—C12—C11	179.6 (2)	C30—C29—C33—C32	1.8 (4)
C10—C11—C12—O1	−179.12 (19)	C28—C29—C33—C32	−178.8 (2)

### Hydrogen-bond geometry (Å, º)

**Table:**

*D*—H···*A*	*D*—H	H···*A*	*D*···*A*	*D*—H···*A*
O3—H3*A*···N2	0.89	2.00	2.888 (3)	173
O3—H3*B*···N1^i^	0.89	2.05	2.940 (3)	173

Symmetry code: (i) −*x*+1, −*y*+1, −*z*+1.
